# Commentary: which child obesity definitions predict health risk?

**DOI:** 10.1186/s13052-017-0337-0

**Published:** 2017-02-04

**Authors:** T. Lobstein

**Affiliations:** 1 World Obesity Federation, 107 Gray’s Inn Road, London, WC1X 8TZ UK; 2 Public Health Advocacy Institute of Western Australia, Curtin University, Bentley, Perth, WA Australia

**Keywords:** Obesity, Adiposity, Risk factors, Epidemiology, Screening

## Abstract

This Commentary considers the different definitions of child overweight and obesity, and reflects on the findings of the paper by Valerio et al. in this issue of the journal.

## Commentary

An ideal measure of child obesity should be accurate in its estimate of body fat; easy to obtain in terms of time, cost and acceptability to the child; and be widely accepted with well-documented, published reference values [1]. In addition any definition of obesity and overweight should be closely associated with actual risk of dysfunction. Sadly, no existing measures or definitions satisfy all these criteria.

The direct measurement of body composition requires techniques such as underwater weighing, magnetic resonance imaging, computerised axial tomography scans or dual energy X-ray absorptiometry. These are impractical for everyday purposes. Less accurate but easier to measure are waist, hip and other girth measurements, skin fold thickness and total body weight. Body weight can be adjusted for height to provide an indirect indicator of adiposity, the well-known Body Mass Index (BMI). Although it has its shortcomings, BMI has been increasingly accepted as a valid indirect measure of adiposity in school-age children and adolescents for survey purposes [2].

How do we interpret the BMI obtained from a child? Among adults, ‘obesity’ is generally defined as a BMI greater than 30 kg/m2, and ‘overweight’ greater than 25 kg/m2 (or sometimes between 25 and 30 kg/m2). These cut-offs for adults are somewhat arbitrary and possibly not appropriate for some sections of the adult population, such as people with Southern Asian backgrounds [3]. Although intended to be related to health risk, it is likely that different disease conditions (e.g. type 2 diabetes, non-alcoholic fatty liver disease, orthopaedic disorders) may have different BMI cut-offs to define given levels of risk. There is no simple gold standard against which the best BMI cut-offs can be defined.

For children there are further difficulties. Healthy children show significant fluctuations in the relationship between weight and height as they grow through infancy and childhood. Charts showing BMI for healthy children by age indicate an initial rapid rise in the first year, a subsequent decline for the next 5 years, and then a slow rise to adulthood. Using BMI of 25 and 30 to define thresholds for overweight and obesity would be misleading.

An expert panel, convened by the International Obesity TaskForce (IOTF) used surveys of children in six countries, with BMI for age and gender charts and plotted equivalent centile curves that passed through the adult cut-off points of BMI 25 and 30 at age 18. The resulting set of age- and gender-specific BMI cut-offs for children was published in 2000 and expanded in 2012 to include equivalents for adult BMIs of 35, 27, 23, 18.5, 17 and 16 kg/m2 [4].

In 2008, the World Health Organization published a set of growth standards obtained from healthy breast-fed babies and infants up to 5 years old surveyed in their Multicentre Growth Reference Study [5]. These ‘standard’ charts were extended from age 5 years to age 19 years using adjusted USA survey data, and the results termed ‘reference’ charts. For children under 5 years old, the WHO proposed cut-offs for ‘at risk of overweight’, ‘overweight’ and ‘obesity’ defined as +1SD, +2SD and +3SD of the standard distribution respectively. For children over the age of 5 years the WHO recommends cut-offs for ‘overweight’ and ‘obesity’ at +1SD and +2SD of the reference distribution respectively.

These two different approaches provide different BMI cut-off thresholds. There is no simple method for reading across from one to the other and in surveys of child populations it is generally recommended that researchers report their results using both the WHO and the IOTF criteria for overweight and obesity, to allow comparability with other surveys [6].

In the paper in this journal by Valerio et al., both the IOTF and WHO categories have been used, along with a set of cut-offs using Italian national data (ISPED). They have compared the results in terms of their sensitivity and specificity for identifying clustered cardiometabolic risk factors in the children. This is a valuable method for deciding which of the cut-off criteria are best for predicting disease risk, but it opens some more questions in turn.

Valerio et al. find the IOTF and ISPED criteria showed higher specificity (i.e. correctly excluding children without cardiovascular risk factors) while the WHO criteria had higher sensitivity (correctly including children with such risk factors). This is not surprising, as the WHO cut-off is generally lower than the IOTF and ISPED cut-offs and will include a larger proportion of the general population. The effect is that the IOTF and ISPED cut-offs will miss some children with risk factors, while the WHO cut-offs will include more children without risk factors.

How do we decide? There are several theoretical methods for choosing an optimum for both sensitivity and specificity, such the Youden Index [7], or rules of thumb, such as finding the cut-offs that have the highest sensitivity with 95% specificity [8]. This would imply that a new set of overweight and obesity cut-offs should be devised which maximise the sensitivity and specificity, at least for cardiovascular risks.

This is unlikely to happen. Furthermore, simply optimising sensitivity and specificity would ignore the differential weighting that might need to be given to the cost or penalty arising from missing positive cases versus the cost or penalty arising from treating false cases. At first glance, the IOTF *overweight* criteria show the highest sensitivity and near-highest specificity, of the three cut-off approaches compared. The WHO *obesity* criteria had the best sensitivity but the worst specificity. If over-treatment is a problem, then the IOTF or ISPED criteria are preferred. If it is important not to miss cases, and if over-treatment is acceptable, then the WHO criteria may be preferred.

In clinical practice, other assessments will be made before deciding on treatment. In epidemiology, the different cut-off criteria will provide different predictions of future disease risks in the population, and generally the WHO approach will give the highest estimates for potential disease. In screening groups of children, the preference might be for the WHO criteria, but this will inevitably include more false positive cases. Staff conducting screening using the WHO criteria should be aware that many children defined by WHO as obese may not require referral for cardiovascular assessment, with its associated risks of creating anxiety for the child and their family.
